# Gender composition of pairs influences joint action effect

**DOI:** 10.3389/fpsyg.2023.1122427

**Published:** 2023-02-23

**Authors:** Marco Fabbri, Monica Martoni, Alessia Beracci, Lorenzo Tonetti, Vincenzo Natale

**Affiliations:** ^1^Department of Psychology, University of Campania Luigi Vanvitelli, Caserta, Italy; ^2^Department of Medical and Surgical Sciences, Alma Mater Studiorum, University of Bologna, Bologna, Italy; ^3^Department of Psychology “Renzo Canestrari”, University of Bologna, Bologna, Italy

**Keywords:** joint action, sex composition, flanker effect, Navon effect, sex difference, social cognition

## Abstract

Research on joint action has demonstrated that individuals are sensitive to a coactor’s attentional relation to jointly attend stimuli. It has also been suggested that some features are necessary to resolve the discrimination problem (i.e., self-own and other-own actions). In the present study, we aimed to test whether the gender composition of interacting pairs modulated the joint action effect. Same- (female-female or male-male) and mixed- (female-male) gender pairs performed a joint version of flanker tasks in Experiment 1 (90 participants, 50% males), while in Experiment 2 (154 participants, 50% males) Navon tasks were performed. In Experiment 1, a higher joint flanker effect in same-gender pairs than in mixed-gender pairs, and this joint effect was similar to the classical flanker effect reported by males and females in a classical procedure of the task (70 participants, 50% males). In Experiment 2, the same-gender pairs reported a joint Navon effect, which was reversed in mixed-gender pairs. In conclusion, our findings support how the gender composition of interacting pairs plays a role in joint attentional tasks.

## 1. Introduction

In the past two decades, cognitive science has begun to consider social influences on cognitive processes, especially when people jointly perform the same task [for a review, see, Sebanz et al. (2006); Dolk et al. (2011, 2014a)]. Using a joint (or social) Simon task (Knoblich and Sebanz, 2006; Sebanz et al., 2006; Dolk et al., 2011, 2014a), two participants, sitting next to each other, displayed a joint Simon effect (JSE) in similar way to a classical Simon effect (SE; Simon, 1969, 1990). Taking into account that the SE reflects a stimulus-response congruency effect (i.e., when there is a spatial correspondence between the spatial position of the target and the spatial position of the response key, the responses are faster than those when there is a spatial incongruency between target and response key; Kornblum et al., 1990), the JSE indicates that both actors represent their own and another person’s action, as well as the representation of the spatial dimension of the responses, introducing a stimulus-response congruency effect (Sebanz et al., 2003). In other words, for the actor, sitting on the left side, all his/her own targets presented on the left space are represented as compatible trials (i.e., spatial congruency between target and response key), while all targets presented on the right space (which is the space of the co-author) are represented as incompatible trials (i.e., spatial incongruency between target and response key). Although the joint Simon task is essentially a go/no-go task (i.e., each participant with a single response has to decide whether to respond to his/her assigned target or to withhold any response), the JSE could be evidence of a dedicate social perception-action mechanism: with a co-representation of another’s task rule/instruction, participants take the active response of their co-actor into consideration when selecting their own responses, and thus in incompatible trials a response selection conflict arises (Sebanz et al., 2003, 2006; Knoblich and Sebanz, 2006; Tsai et al., 2006). This action (or task) co-representation view suggests that each participant has a competition between their own and the other’s response [Sebanz et al., 2003, 2006; Knoblich and Sebanz, 2006; Tsai et al., 2006; Yamaguchi et al., 2017; for a review see Sebanz and Knoblich (2009)].

Intriguingly, the JSE has been also reported in the presence of various dynamic non-human co-actors, such as a Japanese waving cat, suggesting that the joint effect can be induced by any dynamic event (Dolk et al., 2013). These findings indicate that the JSE can be induced by any attention-grabbing event, which facilitates the discrimination between its cognitive representation and the representation of the participant’s own action [Dolk et al., 2013; see also, Liepelt et al. (2011), Pfister et al. (2014), Sellaro et al. (2015), Klempova and Liepelt (2016)]. Even if the spatial response coding account can explain the JSE as matching (or mismatching) between spatial stimulus location and spatial response location (Guagnano et al., 2010; Dittrich et al., 2012, 2013; but see Welsh et al. (2013) for different results) in these conditions, a possible explanation of the JSE is the referential coding account (Dolk et al., 2011, 2013), based on the general assumptions of ideomotor theory [for a review see Stock and Stock (2004)] and Theory of Event Coding (TEC; Hommel et al., 2001; Hommel, 2009, 2011) in particular. TEC assumes that action and perception are tightly linked together, and individuals represent self-produced actions and other perceived events through codes of the effects their actions usually produce. Accordingly, the perception of alternative action or object events representing the same or similar feature codes produces an action selection conflict between actions trigged externally (by another actor) and activated internally. This conflict can be resolved by emphasizing (i.e., intentional weighting principle; Memelink and Hommel, 2013) an action feature, thus better discriminating between self-own and other-own actions [i.e., referential coding; Hommel et al., 2001; Dolk et al., 2013; for a review see, Dolk et al. (2014a)]. Thus, it is possible to expect changes of the JSE related to the perceived similarity between actor and co-actor (Müller et al., 2011; McClung et al., 2013). For example, when group membership (i.e., in-group vs. out-group members) was considered, Müller et al. (2011) found a social Simon effect when white students performed the task with a white left hand displayed on the screen while this effect disappeared when they interacted with a black out-group member, suggesting an action co-representation only when both actor and co-actor belonged to the same group. According to this assumption, gender composition of pairs in joint tasks should affect the joint effect, given that gender is one of the most salient types of social categorization since it is one of the most basic and immediately available social factors that is activated automatically for the construction of social identity and group membership (e.g., Tajfel and Turner, 1979; Stangor et al., 1992; Powlishta, 1995). Mussi et al. (2015) were the first to reveal this gender composition effect with a larger JSE for the same-gender (i.e., female-female or male-male) pair than mixed-gender (i.e., female-male or male-female) pairs showed. These findings indicated that gender, which is automatically accessible, increased the conflict discrimination between self-own and other-own actions in a same-gender pair [for similar results see also, van der Weiden et al. (2016)]. Thus, gender information could influence the joint selective attention. However, van der Weiden et al. (2016) reported a general gender effect on RTs, with faster responses to congruent trials for men independently from co-actor’s sex. This finding limits the influence of gender composition of pairs on joint selective attention, given that gender effect on SE can mask the influence. In addition, van der Weiden et al. (2016) suggested that their results should be replicated in future research because their investigation was the only study, which replicated and extended previous findings provided by Mussi et al. (2015), and the interpretations about the sex of participants in influencing the joint action effect should be taken with caution. However, both Mussi et al. (2015) and van der Weiden et al. (2016) suggested that the gender composition of pairs in joint action paradigms is relevant to better understand the underlying mechanisms of and implications for social interaction. Finally, alternative interpretation (e.g., spatial response coding account; Dittrich et al., 2012, 2013, 2017) for the JSE has been challenged the social nature of the joint Simon task, while different paradigms (e.g., joint Flanker or Navon tasks; Böckler et al., 2012; Dittrich et al., 2017, see below) have been proposed to better understand the social mechanism of joint action.

Interestingly for the purpose of the present study, a growing body of literature shows gender differences for the ability to prioritize the processing of task-relevant information and to filter out distracting task-irrelevant information that might trigger wrong decision [for a review see for e.g., Lee and Choo (2013)]. Using a flanker paradigm, in which participants are generally instructed to attend and respond to centrally presented stimuli (e.g., H) while ignoring nearby flanking stimuli (e.g., S; Eriksen and Eriksen, 1974), it has been observed that females had slower response times (RTs) for incompatible trials (i.e., the central target H is associated with a specific key while the surrounding flankers S are associated with another key) and made more errors, suggesting a higher susceptibility to irrelevant stimuli more than males (Bayliss et al., 2005; Stoet, 2010), demonstrating differential conflict monitoring (Clayson et al., 2011). At the same time, gender differences have been observed when the focus of the attention is shifted from global to local processing of the visual stimuli [for a review see, Herrera et al. (2019)]. In the Navon task (Navon, 1977, 2003) with hierarchically-organized objects, such as large letters (global level; e.g., H) composed of small letters (local level; e.g., h), an advantage or precedence in detecting a target at the global level has been reported, and this advantage is larger for males than females (Navon, 1981; Razumnikova and Volf, 2011). In addition, it has been also reported a congruency effect at local level (Navon, 1977; Kimchi, 1992), that is faster RTs when there is a congruency at global and local levels (e.g., a big H composed of small Hs) respect to there is an incongruency (e.g., a big H composed by small Ss). In this case, the attending to local level processing is easier for women than for men (Müller-Oehring et al., 2007). It is worth noting that both tasks are used in the joint paradigm, suggesting that these cognitive tasks are eligible for examining joint action (Atmaca et al., 2011; Böckler and Sebanz, 2012; Böckler et al., 2012; Dolk et al., 2014b; Dittrich et al., 2017; Fabbri et al., 2017, 2018a,b; Peterburs et al., 2017).

The aim of the present study was to assess how gender information of co-actors influenced the joint selective attention measured in a joint flanker (Experiment 1) and joint Navon (Experiment 2) tasks. We expected to find joint flanker and Navon effects in same-gender pairs, while these effects should disappear in mixed-gender pairs, given that in this latter situation the (diverse) gender information should be sufficient to resolve the action selection conflict between self-own and other-own action turn. In addition, in the Experiment 1, we expected a larger joint flanker effect in female-female pairs than that in male-male pairs, because of females are more prone to process task-irrelevant information than males in a flanker task (Stoet, 2010, 2017). In a similar way, in the Experiment 2, we also expected a larger global advantage in male-male pairs than female-female pairs, whereas the opposite was expected for local processing, because of gender differences in global and local processing (Roalf et al., 2006; Pletzer, 2014).

## 2. Experiment 1

Atmaca et al. (2011) found a joint flanker effect in a go/no-go version of the Eriksen task. Specifically, the joint flanker effect revealed that participants were slower in detecting their own targets surrounded by flankers which were potential targets for their co-actors, compared to stimuli with flankers being part of identical, compatible, and neutral trials. This joint effect was found with a non-human co-actor (a Japanese waving cat), supporting the notion that the joint condition increased the impact of that rule on performance probably because the actor drew attention to it (Dolk et al., 2014b). A person or an inanimate object represented an attention-grabbing event which required participants to discriminate between self-own and other events.

Adopting a procedure used in previous studies (van der Weiden et al., 2016), in this Experiment, in which a joint Eriksen flanker task was used (Atmaca et al., 2011), we tested how the gender of actor and co-actor influenced the joint flanker effect (JFE). Specifically, we expected a JFE in same gender (actor-co-actor gender: female-female and male-male) compared to mixed gender (actor-co-actor gender: female-male and male-female). This finding was expected because in female-female or male-male pairs, the (same) gender of a co-actor increased the perceived similarity to the actor (such as in-group membership; Müller et al., 2011), and therefore representations should become more similar and more difficult to distinguish in the turn-taking response setting. Considering that females are more prone to interference than males in a flanker task (e.g., Stoet, 2010, 2017), it could be possible to find a larger joint flanker effect in female-female pairs than in other types of pairs. To assess this gender difference in selective attention, a sample composed of males and females also undertook a standard two-choice flanker task in which each participant performed lateralized key presses in response to the four target stimuli. This condition also allowed us to ensure that the materials used in the joint/solo conditions yielded a standard flanker effect (following the procedure of Atmaca et al., 2011) and to compare the standard flanker effect with joint and solo flanker effects for both genders.

### 2.1. Materials and methods

#### 2.1.1. Participants

Ninety university students, 45 of whom were males and 45 were females, took part in Experiment 1. From these participants we created 28 same-gender (14 male-male and 14 female-female) and 17 mixed-gender pairs. The mean age was 23.16 years (SD = 6.29 years), which did not differ between different types of gender pairs [male-male: 22.46 ± 3.69 years; female-female: 22.82 ± 6.09 years; mixed-gender: 24.00 ± 8.00 years; *F*(2,87) = 0.51, *p* = 0.60, *η^2^_*p*_* = 0.01], or between males (22.91 ± 4.99 years) and females [23.40 ± 7.42 years; *t*(88), −0.37, *p* = 0.72, Cohen’s *d* = −0.08]. All participants performed both the joint and the individual conditions. All participants had normal or corrected-to-normal vision. Handedness was defined by asking participants which hand they usually preferred to write with (Corey et al., 2001); 90% of participants reported to be right-handed. Depending on handedness, participants sat on the left or right chair in order to prevent involuntarily interference in their partner’s response. All participants were unaware of the purpose of the experiment.

Seventy university students were also recruited to perform a standard flanker task. Half of the participants were males, and the mean age of the sample was 21.79 years (SD = 2.30 years). An age difference between males (22.54 ± 3.01 years) and females (21.03 ± 0.71 years) was found, *t*(68) = 2.90, *p* < 0.005, Cohen’s *d* = 0.69. However, we did not find any age difference in men [*t*(78) = 0.39, *p* = 0.70, Hedges’ *g* = 0.09] and in women [*t*(78) = 1.88, *p* = 0.06, Hedges’ *g* = 0.42] between both samples, and thus male and female mean age was similar in both samples. All participants had normal or corrected-to-normal vision; 90% of participants reported that they preferred the right hand for writing and were unaware of the purpose of the experiment.

All participants gave their written informed consent to participate, and the experiment was conducted in accordance with the ethical standards laid down in the 2013 Declaration of Helsinki (World Medical Association, 2013). The study was approved by the Ethics Committee of the Department of Psychology, University of Campania “Luigi Vanvitelli” and agreed with the Ethical Principles of Italian Psychological Association (AIP^1^).

#### 2.1.2. Materials and procedure

The task materials adopted here were identical to those used by Atmaca et al. (2011); see also Fabbri et al. (2018b). Thus, participants were presented with arrays of five letters and were instructed to detect the target letter in the middle position. In the joint and solo conditions, participants responded to two of four target letters which were H and K for one key, and S and C for another key. The letters H, K, S, C, and U served as flankers in order to create four stimulus types: identical (e.g., HHHHH), compatible (e.g., KKHKK), neutral (e.g., UUHUU), and incompatible (e.g., SSHSS). Participants always responded with the index finger of their dominant hand. In the joint condition, two participants were sitting next to each other and each participants received two target letters. In other words, for each participant the joint task was a go (for the two target letters)/no-go (withhold the responses for the two letters of co-actors) task. In the solo condition there was an empty chair beside the single participant who had to respond to the same two target letters responded to in the joint condition. Thus, the solo condition was experienced as a go/no-go task in the same way to joint condition. Each participant responded to the same target pair with the same key throughout the entire experiment, independently from the fact that he/she performed the individual go/no-go task before or after the joint version. Target pairs (H-K vs. S-C) and response keys (left response using the “z” button of standard keyboard, right response using the number “3” button of standard numerical keypad) were counterbalanced across participants. As noted by Prinz (2015) in the flanker task identical and neutral trials can be considered as control conditions, whereas experimental conditions were provided by compatible and incompatible trials. Indeed, in compatible trials flankers were physically different from the target but required the same response category, whereas in incompatible trials flankers were different from the target, physically and categorically. Thus, we calculated the Flanker Effect (FE) as the difference between RTs of incompatible and compatible trials for joint (JFE) and solo (SFE) conditions. A positive value indicated the presence of the interference (or flanker) effect, while a negative value indicated a reversed flanker effect.

The stimuli presentation and response collection in the Flanker task were controlled *via* computer using the software package E-Prime version 2.0. A white fixation cross was presented for 500 ms in the centre of the black screen with a size 40 Times New Roman font. A blank screen was then presented for 500 ms and was followed by the centrally positioned white stimulus array (size 40 Times New Roman font). After a response key was pressed or 1,000 ms had elapsed, the stimulus array disappeared from the screen and, after a 1,000 ms interval, the next trial started. This trial procedure was identical in both the joint and solo conditions; in both conditions all participants received the instruction to respond to their own target letters and to avoid responding (and thus wait for a new trial for solo condition especially) for all no-go trials (i.e., trials in which a target letter for the co-actor in the joint condition or a non-target letter in the solo condition was presented). For example, whether in the joint condition an actor was instructed to respond to H and K and a co-actor was instructed to respond to S and C, the presentation of target letter S was a no-go trial for one participant and a go trial for another participant. In the solo condition, this trial was a no-go trial for the single actor. However, in both conditions participants were requested to respond as quickly and as accurately as possible for all go trials. In both the joint and solo conditions, there were 48 trials (24 go-, and 24 no-go trials) in which all four trial types occurred equally often. In each experimental block, the trials were presented in random order. In the two-choice condition, participants responded to both pairs of target letters with left and right key presses, according to the same task procedure described above. For example, the left key was pressed to detect H and K target letters and right key was pressed to detect S and C target letters, as described for joint and solo conditions, for a single participant. The combination of target pairs and response keys were counterbalanced across participants. All participants were trained with 16 trials, and feedback on their response speed and accuracy was provided. If participants in the two-choice task, or one or both co-actors in the other two conditions requested further training, the session was performed again before the experimental session.

When a pair of participants arrived at the laboratory, they were verbally informed that they would be performing in two different conditions, acting alone in one condition and taking turns with a second actor in the other condition. In this experiment, we did not use any confederate but both participants responded to assigned two target letters with relative response key, acting as actor and relative co-actor within each pair. Thus, we created four types of pairs according to the gender of the actor (male or female) and the gender of the co-actor (male or female), determining male-male, male-female, female-male and female-female pairs (in similar way to van der Weiden et al., 2016). In order to control for target pairs and response buttons, the following procedure was followed: one participant remained in the room to perform the solo go/no-go flanker task, whereas the other participant was taken into another room where several questionnaires were administered. The two participants then performed the joint flanker task together, and at the end the same questionnaires were administered to the member of the pair who had started with the individual version of the task, whereas the other participant remained seated in order to perform the individual go/no-go Flanker task. At the end of this procedure, both participants were debriefed.

#### 2.1.3. Data analysis

All analyses were performed using the software SPSS version 20.0 (IBM Corp.). The mean reaction times (RTs) were computed for trials in which participants had responded correctly. For joint and solo conditions, all RTs deviating ± 2.5 SD from the mean were excluded and considered as outliers (overall 2.52% of trials). In similar way, for standard two-choices task we computed the mean RTs for correct trials, and all RTs deviating ± 2.5 SD from the mean were excluded and considered as outliers (overall 5.03% of trials). Taking into account that in the joint condition, the mean accuracy was equal to 96.95% (SD = 4.18%), it was equal to 98.02% (SD = 2.85%) in the solo condition, and the accuracy in inhibiting participants’ response to no-go trials in the individual condition was equal to 97.55% (SD = 3.56%), we decided that accuracy was not further analysed because, in general, performance accuracy was high. Overall, in standard two-choices flanker task condition, performance accuracy (*M* = 94.97%; SD = 4.08%) was high. Accuracy remained high for males (*M* = 94.70%; SD = 3.96%) and females (*M* = 95.24%; SD = 4.24%), and no significant gender difference was found, [*t*(68) = −0.55, *p* = 59, Cohen’s *d* = −0.13] Thus, accuracy was not further analysed.

In order to capture the flanker effect (FE), we calculated the RT difference between incompatible and compatible trials, in joint (i.e., JFE), solo (i.e., SFE), and standard (i.e., FE) conditions. Thus, positive differences indicated an interference of incompatible trials respect to compatible trials, suggesting a flanker effect. A positive JFE reflected an interference induced by social context, whereas a positive SFE represented the adoption of specific stimulus-response rule, as postulated by Dolk et al. (2014b).

As regard joint and solo conditions, a mixed ANOVA with Condition (joint vs. solo), as within-subjects factor, and with Sex (male vs. female) and Co-actor’s Sex (male vs. female), as between-subjects factors, was performed on FE, defined by JFE and SFE. As regards standard condition, a between-subjects *t*-test on FE was performed, in order to assess any gender differences in the two-choice Flanker task.

### 2.2. Results

#### 2.2.1. Joint and solo conditions

Table 1 reports descriptive data for RTs and accuracy (defined as number of errors) for all flanker types in both joint and solo conditions for each gender pair.

**TABLE 1:** The means (and relative SDs) of response times (RTs), in ms, and number of errors for each flanker type in joint and solo conditions are displayed for each type of sex pair.

		RTs				Number of errors			
Actor’s sex	Co-actor’s sex	Compatible	Identical	Incompatible	Neutral	Compatible	Identical	Incompatible	Neutral
**Joint task**									
Male	Male	419 (93.10)	408 (60.95)	447 (80.07)	424 (74.93)	0.11 (0.32)	0.11 (0.32)	0.25 (0.52)	0.32 (0.72)
	Female	436 (94.06)	437 (82.19)	454 (74.99)	458 (109.85)	0.06 (0.24)	0.24 (0.44)	0.06 (0.24)	0.18 (0.39)
Female	Male	448 (71.97)	462 (90.62)	461 (63.35)	456 (75.59)	0.00 (0.00)	0.12 (0.33)	0.41 (0.62)	0.00 (0.00)
	Female	412 (41.88)	419 (51.99)	453 (37.21)	427 (42.35)	0.25 (0.44)	0.18 (0.39)	0.18 (0.39)	0.32 (0.48)
**Solo task**									
Male	Male	434 (73.52)	414 (63.50)	448 (68.79)	438 (67.71)	0.04 (0.19)	0.14 (0.36)	0.07 (0.26)	0.14 (0.45)
	Female	435 (56.48)	441 (57.70)	467 (58.16)	443 (56.05)	0.24 (0.44)	0.24 (0.44)	0.12 (0.33)	0.24 (0.44)
Female	Male	451 (73.94)	467 (69.56)	476 (77.26)	463 (55.86)	0.18 (0.39)	0.06 (0.24)	0.06 (0.24)	0.12 (0.33)
	Female	422 (49.52)	414 (41.92)	438 (40.41)	434 (43.99)	0.21 (0.50)	0.07 (0.26)	0.07 (0.26)	0.04 (0.19)
**Two-choice task**									
Gender	Male	486 (60.27)	472 (74.53)	507 (65.57)	495 (75.59)	0.51 (0.70)	0.60 (0.78)	0.94 (0.87)	0.49 (0.82)
	Female	511 (68.69)	514 (78.31)	556 (79.53)	525 (72.88)	0.54 (0.66)	0.57 (0.85)	0.71 (0.93)	0.46 (0.66)

In addition, the table reports mean RTs and number of errors (with relative SDs) for each flanker type of two-choice flanker task separately for males and females.

**FIGURE 1:**
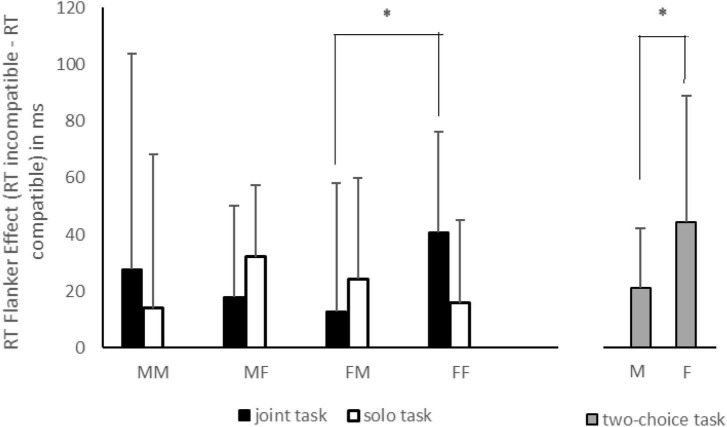
The flanker effect (FE), reported as the difference between response times (RTs) of incompatible and compatible trials for each sex pair (MM, male-male; MF, male-female; FM, female-male; FF, female-female) in joint (black histograms) and solo (white histograms) tasks. The gray histograms represent the FE for males and females in a classical two-choice task. The asterisks indicate the significant comparisons. The bars represent the standard deviations.

The mixed ANOVA did not reveal any significant main effects (*Fs* < 1.00, *ps* > 0.36, and *η^2^_*p*_* < 0.01) or double interactions (*Fs* < 1.00, *ps* > 0.60, and *η^2^_*p*_* < 0.003). The only significant result was the triple interaction between factors [*F*(1,86) = 5.69, *p* < 0.05, *η^2^_*p*_* = 0.06], as displayed in Figure 1. Using Tukey’s *post-hoc* test for unequal sample size, we observed a larger JFE for female-female pairs than that for female-male pairs in the joint task (*p* < 0.05), while a non-significant difference of JFE was found when comparing male-male and male-female pairs (*p* = 0.61). As regards SFE, no significant comparisons were found among sex pairs (*ps* > 0.20 for both comparisons).

#### 2.2.2. Standard two-choice flanker task condition

Table 1 reports descriptive data for RTs and accuracy (defined as number of errors) for all flanker types of the classical Flanker task, for males and females. The between-subjects *t*-test on standard FE showed a significant difference with a larger FE in females than in males, [*t*(68) = −2.36, *p* < 0.05, Cohen’s *d* = −0.56], as displayed in Figure 1.

#### 2.2.3. Flanker effect comparisons

As stated above, the standard two-choice Eriksen task could be used as a frame of reference in order to assess the similarity (or the difference) between JFE/SFE and the standard FE. Thus, we decided to perform a set of *t*-tests comparing the JFE and SFE, obtained in same-gender or mixed-gender pair conditions, to the standard FE shown in the two-choice task, separately for gender. For men, the mean standard FE was equal to 21.12 ms (SD = 40.60 ms), whereas for women the standard FE was equal to 44.40 ms (SD = 42.11 ms). Taking into account that we performed 8 *t*-test comparisons (4 for each gender), we decided to use a Bonferroni correction of *p* value (0.05/8 = 0.006). As regards male actors, none of the comparisons were significant (*ts* < 1.10, *ps* > 0.30, and Hedges’ *g* < 0.30), indicating that JFE (with other males and females) and SFE did not deviate from the standard FE of the two-choice task. A similar result was also found for female actors (*ts* < −2.50, *ps* > 0.05, and Hedges’ *g* < 0.75) with the sole exception of SFE [*t*(61) = −3.05, *p* < 0.005, and Hedges’ *g* = 0.77]. On the whole, these results confirmed the gender difference in JFE and SFE and, at the same time, the reliability of materials used in the social condition, yielding a similar FE between joint/solo and standard tasks.

### 2.3. Discussion

Experiment 1 was aimed at investigating whether the JFE could be influenced by the (same or mixed) gender of actor and co-actor in a pair during joint and solo tasks. As a control condition, we asked men and women to perform a standard two-choice flanker task. As displayed in Figure 1, the main results were: a larger JFE for same-gender pairs than for mixed-gender pairs, and this was particularly relevant for females; no significant differences between same- and mixed-gender pairs were found for the SFE. In the standard Eriksen task, the FE for women was higher than that found for men, suggesting a sex difference in interference effect; the JFE and SFE found were similar to the standard FE with the only exception being for females in the solo condition.

Extending the findings of previous works (Mussi et al., 2015; van der Weiden et al., 2016), our experiments demonstrated that the co-actor’s sex played a relevant role in modulating the action interference effect. Thus, our data seem to support the notion that gender may constitute an immediate information (Powlishta, 1995) to distinguish from self-own and other-own responses, in line with studies displaying a JSE when actors interacted with other “in-group” participants (Müller et al., 2011; McClung et al., 2013). Thus, our data could indicate that the co-actor’s gender, especially in mixed-gender pairs, might act more as a distinctive feature in order to discriminate from the self-own response to other events (Dolk et al., 2011, 2013, 2014a,b), driving a larger suppression of the location response code and a reduction of the effects exerted by compatible and incompatible flankers, probably due to an automatic activation of gender categorization (Ito and Urland, 2003). In same-gender pairs, the co-actor’s sex became a salient feature and increased the need to make the internal representation of self-own response more distinctive. This, in turn, induced a greater response discrimination with an increase in FE in the joint paradigm. In addition, we found a larger JFE in female-female pairs compared to that exhibited by male-male pairs, suggesting that women were more distracted by irrelevant flankers, potentially demonstrating gender differences in conflict monitoring (Botvinick et al., 2001; Bayliss et al., 2005; Stoet, 2010, 2017). This assumption was based on gender differences found in the standard flanker task. At the same time, this higher distractibility for women compared to men could also reflect a greater “sensibility” on the part of women when processing the social context during an interaction with other people. Indeed, females are expected to be friendlier and more communal than males in society (e.g., Eagly and Wood, 1999; Witt and Wood, 2010). Thus, the Sex effect on JFE could be related not only to the gender differences in processing irrelevant stimuli such as flankers, but also to gender differences in inhibiting the social processing, given that in the joint Eriksen task the co-actor was an attention-grabbing event which impaired females’ ability to focus their selective attention on the central target (Dittrich et al., 2017; Fabbri et al., 2018b). Although previous arguments seemed to be speculative, the lack of significant comparisons between JFE or SFE and standard FE (with the sole exception of SFE in females) showed that the materials used in all tasks were reliable, given that the stimuli induced and exerted similar response-related processing with similar flanker effects between conditions.

However, this first study did not rule out the possibility that the nature of the flanker task induced a specific cognitive strategy in participants’ performance of the task. Indeed, the JFE was larger in female-female pairs than in male-male pairs, and thus it could be the tendency for females to have lower inhibition control in flanker task (Botvinick et al., 2001; Bayliss et al., 2005). Indeed, in the classical Eriksen task, we found larger FE for females than males, and this FE was “summed” in female-female pairs. Thus, in Experiment 2 we tried to address these findings using a joint Navon task. Firstly, this task is another measure of selective attention, given that it requires the processing of specific types of information (Stoet, 2017). Secondly, this joint task maximizes the relevance of task instruction, given that the joint Navon effect (or JNE) arises when both actors receive different attentional instructions. Finally, the classical version of the Navon task has clearly demonstrated gender differences (e.g., Halpern, 2012; Pletzer, 2014), with an advantage of men for global processing and an advantage of women for local processing.

## 3. Experiment 2

Böckler et al. (2012); see also Böckler and Sebanz (2012); Fabbri et al. (2017, 2018a) required two participants, sitting next to each other, to perform a two-choice task on the identity of Navon letters (i.e., S and F for an actor and H and O for the co-actor). The letters assigned to the two participants were never intermixed and participants performed a go/no-go task. The Navon letters could either be congruent (linked to the same response: a large S composed of small Ss) or incongruent (linked to different responses: a large S composed by small Fs). In different experimental blocks, the authors required participants to focus either on the global or local features of letters. Crucially, the participants’ task required them to adopt the same focus of attention (e.g., both participants attending to global stimulus features) or a different focus of attention (one attending to the global features and the other attending to local features). The main finding was a slowing of RTs when participants attended to different stimulus features, indicating that actors hold a representation not only of their own but also of the co-actor’s attentional focus (i.e., JNE). This increase in RTs for different foci of attention was found for both congruent and incongruent stimuli as well as when participants attended to global or local stimulus aspects (Böckler and Sebanz, 2012; Böckler et al., 2012), supporting the selection-conflict hypothesis (i.e., the representation of the other’s different task generally increases the difficulty of selecting and maintaining one’s own focus of attention). Although Fabbri et al. (2017, 2018a) confirmed the JNE in different populations and with specific experimental protocol, their results more supported the biased-focus hypothesis (i.e., co-actors are biased toward the focus of the other, shifting, for example, their own local focus toward the other’s global focus). As noted by Böckler et al. (2012), neither of these hypotheses necessarily excludes the other, because they suggested that participants experience a conflict as to which focus to select and shift toward the focus of the other with a slowing-down of responses when attentional foci differ and an increased difference in RTs between congruent and incongruent stimuli.

The aim of this Experiment 2 was to find a larger JNE for same-gender pairs and no (or even reversed) JNE for mixed-gender pairs. Indeed, in the joint condition, a response discrimination problem existed when a different instruction was provided because the decision as to which response to make needed to await the resolution of the slowest completion (among a number of different competing items in event representations) and Navon letters exerted their effect on response competition, with an increase in RTs for incongruent stimuli, suggesting a greater response discrimination. Thus, the gender information provided by the co-actor should induce faster responses in mixed-gender pairs when participants attend to different foci because if the co-actor is of a different gender this should resolve the conflict involved in selecting the adequate focus. Indeed, when a different task instruction (to attend to global features for one and to attend to local features for the other) was provided, it was more probable that the information of (different) gender of participants was used to discriminate between self-own and other events than when both actors attended to same foci. Given that it has been reported a male’s superiority for global processing and a female’s advantage for local processing (Roalf et al., 2006; Pletzer, 2014), a larger JNE for same-gender pairs than mixed-gender pairs should represent a proof of how gender information played a role in social cognition.

### 3.1. Materials and methods

#### 3.1.1. Participants

One hundred and fifty-four university students took part in Experiment 2. None of the participants was involved in the previous experiment. There were 77 males and 77 females. In this way, we created 26 male-male, 26 female-female and 25 mixed-gender pairs. The mean age was 23.31 years (SD = 3.76 years), which did not differ between different types of gender pairs [male-male: 23.35 ± 3.73 years; female-female: 22.88 ± 2.64 years; mixed-gender: 23.72 ± 4.69 years; *F*(2,151) = 0.63, *p* = 0.53, *η^2^_*p*_* = 0.008], or between males (23.56 ± 4.25 years) and females [23.06 ± 3.19 years; *t*(152) = 0.82, *p* = 0.42, Cohen’s *d* = + 0.13]. All participants had normal or corrected-to-normal vision. Handedness was defined by asking participants which hand they usually preferred to write with (Corey et al., 2001); 90% of participants reported to be right-handed. Depending on handedness, participants sat on the left or right chair in order to prevent them from involuntarily interfering with the other’s response. All participants were unaware of the purpose of the experiment and gave their written informed consent to participate. The experiment was conducted in accordance with the ethical standards laid down in the 2013 Declaration of Helsinki (World Medical Association, 2013). The study was approved by the Ethics Committee of the Department of Psychology, University of Campania “Luigi Vanvitelli” and agreed with the Ethical Principles of Italian Psychological Association [AIP(see text footnote 1)].

#### 3.1.2. Material and procedure

The task materials adopted here were identical to those used by Böckler et al. (2012); see also Fabbri et al. (2017, 2018a). Pairs of participants were tested, sitting next to each other in a room. Each participant sat in front of a 22-in. monitor at a viewing distance of 60 cm. The stimuli were large letters (F, H, O, S; 2.3° × 3.8° visual angle) consisting of repeated small letters (f, h, o, s; 0.24° × 0.5° visual angle). Each participant was assigned two target letters, that is, F and S for the participant on the left and H and O for the participant on the right. The letters could be congruent e.g., a large F made up of small Fs or incongruent (e.g., a large F made of small Ss), but the letters of the two participants were never intermixed, creating a go/no-go experimental task. Taking into account the 4 letters and the congruent or incongruent conditions, eight different stimuli were presented equally in a randomized order. As in Experiment 1, the combination between the actor’s and the co-actor’s gender determined 4 types of pairs: male-male, male-female, female-male, female-female (e.g., van der Weiden et al., 2016).

The stimuli presentation and response collection in the joint Navon task were controlled *via* computer using E-Prime 2.0. Each trial started with the presentation of a fixation cross in the center of the screen for 900 ms. Subsequently, a Navon letter appeared at the center of the screen for 200 ms. After a subject responded or 1,100 ms had lapsed, a black screen appeared for a randomized 700–1,000 ms interstimulus interval. The stimuli appeared in a randomized order and the task instruction required participants to respond only to their own target letters (go-trials) by pressing one of two keys (relative to the two target letters) with the index fingers of their left and right hand and to abstain from reacting to their co-actor’s letters (no-go trials). The importance of speed and accuracy were emphasized in the instruction. Responses were collected *via* two button boxes with two horizontally arranged keys. A cardboard box was placed above each participant’s hand in order to prevent him/her from perceiving the other’s responses.

The four experimental blocks were preceded by two practice blocks (for global and local focus, separately). Each experimental block consisted of 48 trials (with 24 go- and 24 no-go trials) and between blocks a short rest period was allowed. Within blocks, congruent and incongruent stimuli were randomized. Half of the trials contained congruent stimuli, while the other half consisted of incongruent letters. Before each block, the task instructions appeared on the screen and clearly indicated the focus of attention to be adopted by each individual. Specifically, the instructions provided four conditions in which both participants had the same global or local focus of attention, or alternately, participants had different attentional foci (e.g., the participant sitting on the left focused on global features while the participant sitting on the right focused on local features). Hence, the combination of one’s own (global vs. local) and the other’s task (same vs. different) appeared in the four blocks. Overall, the experimental session took about 30–40 min.

#### 3.1.3. Data analysis

All analyses were performed using the software SPSS version 20.0 (IBM Corp.). The mean RTs were computed for trials in which participants had responded correctly. All RTs deviating ± 2.5 SD from the mean were also excluded as outliers (overall 3.75% of trials). On the whole, performance accuracy was equal to 96.29% (SD = 3.48%) and therefore was not further analysed, given that it was also positively correlated with mean RTs (*r* = 0.35, *p* < 0.0001), suggesting that there was no speed-accuracy trade-off. A mixed ANOVA was performed on RTs including the variables Task (same vs. different), Focus (global vs. local), and Congruency (congruent vs. incongruent) as within-subjects factors, and the variables Sex (male vs. female) and Co-actor’s Sex (male vs. female) as between-subjects factors. As suggested by Böckler et al. (2012), Fabbri et al. (2017, 2018a), the JNE was shown by faster RTs when same attentional task was implemented than when different attentional task was requested.

### 3.2. Results

Table 2 reports descriptive data for RTs and accuracy for congruent and incongruent stimuli in both the global and local focus of attention, separately for same and different attentional foci for each type of gender pair.

**TABLE 2:** The means (and relative SDs) of response times (RTs), in ms, (upper part) and number of errors (lower part) for congruent and incongruent trials in global and local focus of attention when the same and different attentional tasks were provided are displayed for each sex pair.

		Same task				Different task			
		Global focus		Local focus		Global focus		Local focus	
Actor’s sex	Co-actor’s sex	Congruent	Incongruent	Congruent	Incongruent	Congruent	Incongruent	Congruent	Incongruent
**RTs**									
Male	Male	648 (114)	673 (117)	669 (114)	714 (105)	649 (123)	688 (144)	692 (119)	749 (109)
	Female	687 (136)	734 (185)	682 (127)	728 (136)	653 (138)	697 (148)	704 (158)	742 (114)
Female	Male	779 (204)	788 (179)	762 (143)	781 (132)	720 (152)	747 (137)	763 (140)	792 (121)
	Female	707 (118)	747 (157)	736 (122)	787 (123)	711 (144)	753 (166)	766 (120)	817 (111)
**Number of errors**									
Male	Male	0.38 (0.63)	0.54 (0.78)	0.52 (0.64)	0.67 (1.08)	0.50 (0.98)	0.54 (1.08)	0.48 (0.85)	0.69 (1.00)
	Female	0.36 (0.57)	0.32 (0.56)	0.48 (0.65)	0.48 (0.65)	0.48 (0.65)	0.44 (0.71)	0.52 (0.65)	0.84 (2.01)
Female	Male	0.32 (0.56)	0.64 (0.81)	0.32 (0.56)	1.08 (2.04)	0.40 (0.65)	0.44 (0.71)	0.44 (0.71)	1.04 (2.19)
	Female	0.33 (0.65)	0.38 (0.53)	0.37 (0.72)	0.48 (1.09)	0.37 (0.60)	0.35 (0.48)	0.27 (0.49)	0.63 (0.77)

**FIGURE 2:**
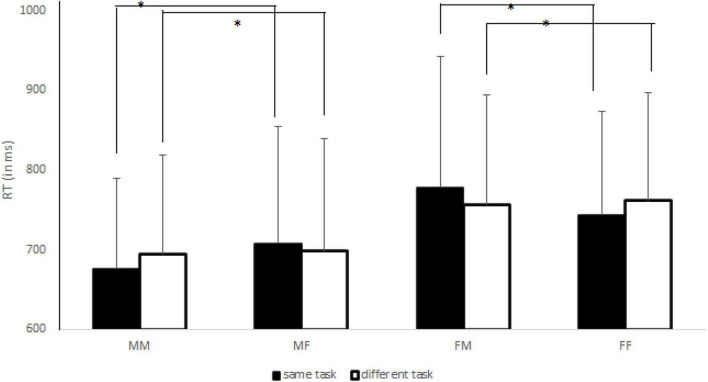
The joint Navon effect (JNE) for each sex pair (MM, male-male; MF, male-female; FM, female-male; FF, female-female) in same (black histograms) and different (white histograms) task blocks. The asterisks indicate the significant comparisons. The bars represent the standard deviations.

The mixed ANOVA showed a Sex effect [*F*(1,150) = 10.56, *p* < 0.005, *η^2^_*p*_* = 0.07], with faster responses performed by males (*M* = 694 ms; SD = 130 ms) than by females (*M* = 760 ms; SD = 142 ms). Classical Navon effects were also found, such as Focus [*F*(1,150) = 27.08, *p* < 0.0001, *η^2^_*p*_* = 0.15] and Congruency [*F*(1,150) = 133.32, *p* < 0.0001, *η^2^_*p*_* = 0.47] effects. The former effect indicated a global preference, that is, faster RTs for detecting global features (*M* = 711 ms; SD = 148 ms) of stimuli than for detecting local features (*M* = 743 ms; SD = 125 ms) of target letters. The latter effect showed faster RTs for congruent (*M* = 708 ms; SD = 136 ms) than incongruent (*M* = 746 ms; SD = 137 ms) conditions. No other main effects were significant (*Fs* < 1.00, *ps* > 0.70, and *η^2^_*p*_* < 0.0001). The analysis showed a significant Task x Focus interaction [*F*(1,150) = 10.22, *p* < 0.005, *η^2^_*p*_* = 0.06]. The *post-hoc* test revealed that when the same attentional task was requested the global precedence was reduced (difference between RT local–RT global = + 12 ms) whereas it was significantly larger when different attentional task was requested (+ 51 ms), with *p* < 0.05 for all comparisons. In addition, we found a significant Coactor’s Sex x Congruency interaction [*F*(1,150) = 4.02, *p* < 0.05, *η^2^_*p*_* = 0.03], suggesting that the congruency effect (difference between RT incongruent–RT congruent trials) was larger for female (+ 45 ms) than for male (+ 32 ms) co-actors, with *p* < 0.05 for all comparisons. Importantly, we observed the significant triple interaction between Sex, Coactor’s Sex and Task [*F*(1,150) = 13.07, *p* < 0.0001, *η^2^_*p*_* = 0.08]. As shown in Figure 2, in same-gender pairs a JNE was found, while in mixed-gender pairs a reversed JNE was observed. In addition, the Sex x Coactor’s Sex x Focus interaction was significant [*F*(1,150) = 4.54, *p* < 0.05, *η^2^_*p*_* = 0.03]. Specifically, we observed a “classical” global preference in male-male (+ 41 ms) and in female-female (+ 47 ms) pairs, while it was significantly reduced in male-female (+ 21 ms) and female-male (+ 16 ms) pairs (*p* < 0.05 for all comparisons). No other significant interactions were found (*Fs* < 3.00, *ps* > 0.09 and *η^2^_*p*_* < 0.02).

### 3.3. Discussion

The aim of Experiment 2 was to test whether the JNE was larger in same-gender pairs respect to mixed-gender pairs in the social Navon task (Böckler et al., 2012; Fabbri et al., 2017, 2018a), while participants either attended to the same aspect of Navon letters (i.e., both attending to the global or to the local features) or attended to different aspects (i.e., one attending to the global and the other to the local feature). As shown in the Figure 2, the gender composition of the pairs interacted with the type of attentional task required of both participants, extending the results found for JSE (Mussi et al., 2015; van der Weiden et al., 2016). Indeed, we found, in same-gender pairs, a significant JNE while, in mixed-gender pairs, a reversed JNE was found. It is possible to posit that the co-actor’s gender was used as a discriminating feature (Powlishta, 1995; Ito and Urland, 2003) to make the internal representation of the participant’s own turn to respond more distinctively especially when different attentional foci were provided by the task instructions, in line with studies displaying JSE when actors interacted with other “in-group” participants (Müller et al., 2011; McClung et al., 2013). Indeed, in the present study, we observed that the global precedence was significantly reduced for mixed-gender pairs whereas it remained reliable in the same-gender pairs, further confirming that the gender of co-actor was a reliable feature in order to resolve the response discrimination problem. This explanation could be supported by the fact that we observed classical Navon effects, such as global preference and congruency effects, indicating the reliability of the Navon task, also in this joint version. Moreover, we found that the global preference was larger in the different than in the same task blocks, suggesting that Navon letters exerted their effect on response competition by activating either the same response that the central target letter was activating (reducing the global preference in the same attentional task) or by activating a competing response (increasing the global preference in the different attentional task), especially for females.

As regards gender difference in the Navon task, we found a general tendency for male participants to respond faster to the Navon letters compared to female participants, while we did not confirm the stronger global advantage in men. For instance, Lee et al. (2012) reported the same behavioral data, probably confirming that males have superior visuospatial abilities compared to females (Halpern, 2012). It is possible, in fact, that the Navon letter task requires visuospatial recognition of patterns rather than language processing, explaining the superiority of male individuals in visuo-spatial tasks (Roalf et al., 2006; Pletzer, 2014). Contrarily from Experiment 1, the general advantage of males in the task did not limit the main results because, as displayed in the Figure 2, the JNE was always found in same-gender pairs while a reversed JNE was always found in the mixed-gender pairs, suggesting that the gender information of own co-actor influenced more the performance than general advantage of men in the Navon task.

## 4. General discussion

The aim of the present research was to assess how gender composition (same-gender vs. mixed-gender) of pairs influences JFE (Experiment 1) and JNE (Experiment 2), using two previously used joint action tasks (Atmaca et al., 2011; Böckler et al., 2012). The results clearly demonstrate that the gender of both actors influenced the joint action effects, given that JFE and JNE appeared in same-gender pairs while they disappeared (or were reversed) in mixed-gender pairs. The presence of another attention-grabbing event induced the tendency to discriminate its cognitive representation from the representation of the participant’s own action. A different gender between participants in a joint task could be considered a distinctive feature to resolve the response discrimination problem, that is, to discriminate between self-generated and other-generated actions. In line with previous results regarding the joint Simon effect (Mussi et al., 2015; van der Weiden et al., 2016), gender may instigate in-group/out-group categorization processes (Tajfel and Turner, 1979; Stangor et al., 1992; Powlishta, 1995; Ito and Urland, 2003; Müller et al., 2011; McClung et al., 2013). Indeed, gender is spontaneously used to categories social partners as in-group or out-group as compared to themselves. This information is used when participants represent their own action and the actions of another person, or, in other words, it is used to discriminate between one’s own turn and the other’s turn in joint tasks (Dolk et al., 2013). This explanation was further confirmed by two additional facts. First, the results of our first experiment resembled those reported by Dolk et al. (2014b) using a non-human co-actor (a Japanese waving cat), thus supporting this data interpretation. Second, the RT pattern of Experiment 2 resembled the RT patterns displayed by Böckler et al. (2012) in Figure 2, 3 of their study (pages 1,407 and 1,409). This similarity could, again, be discussed considering the role of gender composition in interacting pairs as a distinctive feature dimension to resolve the response discrimination problem. Future studies should address in depth the role of gender in modulating other joint action effects.

Even though it was not a direct goal of the present study, in both experiments we found some results related to gender differences in selective attention, measured by flanker and Navon tasks. On one hand, we confirmed that the difficulty reported by women in inhibition function using a go/no-go version of the Eriksen flanker task (Bayliss et al., 2005; Stoet, 2010, 2017), probably reflects gender differences in the general conflict monitoring process (Botvinick et al., 2001). This finding could also be discussed in terms of the sensibility of women in processing the social context in joint paradigm (Eagly and Wood, 1999; Witt and Wood, 2010). On the other hand, we found that males were faster at responding to stimuli than females, suggesting general differences in spatial and verbal abilities (Roalf et al., 2006; Halpern, 2012; Pletzer, 2014), most probably due to the visuospatial nature of the task (Lee et al., 2012), even if we did not confirm the preference of men for global processing (Roalf et al., 2006; Pletzer, 2014). Although the gender difference in the joint flanker task could limit the influence of gender composition of pairs in the joint selective attention, this possibility was excluded in the joint Navon task, which appeared to be more reliable to capture the role of gender composition of pairs in social contexts. Future studies should address gender differences in selective attention using standard and joint versions of flanker (Eriksen and Eriksen, 1974) and Navon (Navon, 1977, 1981, 2003) tasks to deeply understand these effects.

Although our study, through two experiments, had the merit to shed some light on joint action, the present research is not without limitations. Indeed, it has been found that the joint action effect is high when the two co-acting participants are engaged in a positive (Hommel et al., 2009), a cooperative relationship (Ruys and Aarts, 2010; Iani et al., 2011), and/or in interpersonal relationship (Mussi et al., 2015; van der Weiden et al., 2016). These types of information were not controlled for in the present study, despite of large sample in both experiments. At the same time, a cognitive state which induces information integration, such as good mood, should counteract discrimination between actors and co-actors, which in turn should increase the JFE and JNE (e.g., Colzato et al., 2013). Future studies should address these possibilities, using the social paradigms of the present study. Another limit regards the possibility that our participants in the same- and mixed-gender pairs used alternative strategies, such as specific stimulus-response rule or association, in order to perform correctly the task, limiting the impact of the processing of gender similarity or difference as discrimination rule for self-own and other turn to respond (Dolk et al., 2011). Additional studies investigating the social nature of the joint Flanker and Navon effects when two participants interact in the same task are needed.

To sum up, the present findings suggest that it is important to consider the gender composition of interacting pairs when the underlying mechanisms of, and implications for, social interaction are studied, considering the automatic in-group/out-group categorization processes provided by gender. The referential coding and co-representation account seem to be both comprehensive and valid approaches to explain the social nature of joint action paradigm and attentional joint effects. Indeed, our data could be discussed by co-representation account given that this account could predict that same gender (i.e., in-group member) is more likely to be co-represented than other gender co-actor (i.e., an out-group member; Sebanz et al., 2003, 2006; Sebanz and Knoblich, 2009; Atmaca et al., 2011; Böckler et al., 2012). At the same time, our data could be discussed by the referential coding account because it is possible to speculate that women and men represent the different (from themselves) gender of their co-actor and use this information for the representation of self-generated events (Dolk et al., 2013, 2014a,b; Fabbri et al., 2017, 2018a). Thus, the JFE and JNE might be taken as indicators of the similarity between self- and other-generated events, and measures of the degree of self-other integration, particularly in social contexts, with emphasis on more basic features of participants, such as their gender. Future studies should address in deep way which account in a better way explains the joint action effects. Finally, the go/no-go joint paradigms are a valuable educational or rehabilitative tool (Fabbri et al., 2018b).

## Data availability statement

The raw data supporting the conclusions of this article will be made available by the authors, without undue reservation.

## Ethics statement

The studies involving human participants were reviewed and approved by Ethics Committee of the Department of Psychology, University of Campania Luigi Vanvitelli. The patients/participants provided their written informed consent to participate in this study.

## Author contributions

MF, MM, and VN designed the study. MF and AB collected and analysed the data. MM and LT supervised the study. MF wrote the original draft. MM, LT, AB, and VN revised the draft. All authors contributed to the article and approved the submitted version.

## Footnotes

1
https://aipass.org/chi-siamo/#ethical-code

## References

[B1] Atmaca S. Sebanz N. Knoblich G. (2011). The joint flanker effect: Sharing tasks with real and imagined co-actors. *Exp. Brain Res.* 211 371–385. 10.1007/s00221-011-2709-9 21573746 PMC3102196

[B2] Bayliss A. P. Di Pellegrino G. Tipper S. P. (2005). Sex differences in eye gaze and symbolic cueing of attention. *Q. J. Exp. Psychol.* 58A 631–650. 10.1080/02724980443000124 16104099

[B3] Böckler A. Knoblich G. Sebanz N. (2012). Effects of a coactor’s focus of attention on task performance. *J. Exp. Psychol. Hum. Percept. Perform.* 38 1404–1415. 10.1037/a0027523 22409143

[B4] Böckler A. Sebanz N. (2012). A co-actor’s focus of attention affects stimulus processing and task performance: An ERP study. *Soc. Neurosci.* 7 565–577. 10.1080/17470919.2012.682119 22524148

[B5] Botvinick M. M. Braver T. S. Barch D. M. Carter C. S. Cohen J. D. (2001). Conflict monitoring and cognitive control. *Psychol. Rev.* 108 624–652. 10.1037/0033-295x.108.3.624 11488380

[B6] Clayson P. E. Clawson A. Larson M. J. (2011). Sex differences in electrophysiological indices of conflict monitoring. *Biol. Psychol.* 87 282–289. 10.1016/j.biopsycho.2011.03.011 21470571

[B7] Colzato L. S. van den Wildenberg W. P. M. Hommel B. (2013). Increasing self-other integration through divergent thinking. *Psychon. Bull. Rev.* 20 1011–1016. 10.3758/s13423-013-0413-4 23440727

[B8] Corey D. M. Hurley M. M. Foundas A. L. (2001). Right and left handedness defined: A multivariate approach using hand preference and hand performance measures. *Neuropsychiatry Neuropsychol. Behav. Neurol.* 14 144–152. 11513097

[B9] Dittrich K. Bossert M. L. Rothe-Wulf A. Klauer K. C. (2017). The joint flanker effect and the joint Simon effect: On the compatibility of processes underlying joint compatibility effects. *Q. J. Exp. Psychol.* 70 1808–1823. 10.1080/17470218.2016.1207690 27357224

[B10] Dittrich K. Dolk T. Rothe-Wulf A. Klauer K. C. Prinz W. (2013). Keys and seats: Spatial response coding underlying the joint spatial compatibility effect. *Atten. Percept. Psychophys.* 75 1725–1736. 10.3758/s13414-013-0524-z 23896690

[B11] Dittrich K. Rothe A. Klauer K. C. (2012). Increased spatial salience in the social Simon task: A response-coding account of spatial compatibility effects. *Atten. Percept. Psychophys.* 74 911–929. 10.3758/s13414-012-0304-1 22528612

[B12] Dolk T. Hommel B. Colzato L. S. Schütz-Bosbach S. Prinz W. Liepelt R. (2011). How “social” is the social Simon effect? *Front. Psychol.* 2:84. 10.3389/fpsyg.2011.00084 21687453 PMC3110342

[B13] Dolk T. Hommel B. Colzato L. S. Schütz-Bosbach S. Prinz W. Liepelt R. (2014a). The joint Simon effect: A review and theoretical integration. *Front. Psychol.* 5:974. 10.3389/fpsyg.2014.00974 25249991 PMC4155780

[B14] Dolk T. Hommel B. Prinz W. Liepelt R. (2014b). The joint flanker effect: Less social than previously thought. *Psychon. Bull. Rev.* 21 1224–1230. 10.3758/s13423-014-0583-8 24496739

[B15] Dolk T. Hommel B. Prinz W. Liepelt R. (2013). The (not so) social Simon effect: A referential coding account. *J. Exp. Psychol. Hum. Percept. Perform.* 39 1248–1260. 10.1037/a0031031 23339346

[B16] Eagly A. H. Wood W. (1999). The origins of sex differences in human behaviour: Evolved dispositions versus social roles. *Am. Psychol.* 16 143–149. 10.1037/0003-066x.54.6.408

[B17] Eriksen B. A. Eriksen C. W. (1974). Effects of noise letters upon the identification of a target letter in a nonsearch task. *Percept. Psychophys.* 16 143–149. 10.3758/BF03203267

[B18] Fabbri M. Frisoni M. Martoni M. Tonetti L. Natale V. (2017). Synchrony effect on joint attention. *Exp. Brain Res.* 235 2449–2462. 10.1007/s00221-017-4984-6 28509111

[B19] Fabbri M. Frisoni M. Martoni M. Tonetti L. Natale V. (2018a). Influence of time-of-day on joint Navon effect. *Cogn. Process.* 19 27–40. 10.1007/s10339-017-0849-y 29185170

[B20] Fabbri M. Vitale C. Cuoco S. Beracci A. Calabrese R. Cordella M. (2018b). Theory of mind and joint action in Parkinson’s disease. *Cogn. Affect. Behav. Neurosci.* 18 1320–1337. 10.3758/s13415-018-0642-0 30259349

[B21] Guagnano D. Rusconi E. Umiltà C. A. (2010). Sharing a task or sharing space? On the effect of the confederate in action coding in a detection task. *Cognition* 114 348–355. 10.1016/j.cognition.2009.10.008 19914615

[B22] Halpern D. F. (2012). *Sex differences in cognitive abilities*, 4th Edn. New York, NY: Psychology Press.

[B23] Herrera A. Y. Wang J. Mather M. (2019). The gist and details of sex differences in cognition and the brain: How parallels in sex differences across domains are shaped by the locus coeruleus and catecholamine systems. *Prog. Neurobiol.* 176 120–133. 10.1016/j.pneurobio.2018.05.005 29772255 PMC6485927

[B24] Hommel B. (2009). Action control according to TEC (theory of event coding). *Psychol. Res.* 73 512–526. 10.1007/s00426-009-0234-2 19337749 PMC2694931

[B25] Hommel B. (2011). The Simon effect as tool and heuristic. *Acta Psychol.* 136 189–202. 10.1016/j.actpsy.2010.04.011 20507830

[B26] Hommel B. Colzato L. S. van den Wildenberg W. P. M. (2009). How social are task representations? *Psychol. Sci.* 20 794–798. 10.1111/j.1467-9280.2009.02.367.x 19493327

[B27] Hommel B. Müsseler J. Aschersleben G. Prinz W. (2001). The theory of event coding (TEC): A framework for perception and action planning. *Behav. Brain Sci.* 24 849–878. 10.1017/S0140525X01000103 12239891

[B28] Iani C. Anelli F. Nicoletti R. Arcuri L. Rubichi S. (2011). The role of group membership on the modulation of joint action. *Exp. Brain Res.* 211 439–445. 10.1007/s00221-011-2651-x 21472442

[B29] Ito T. A. Urland G. R. (2003). Race and gender on the brain: Electrocortical measures of attention to race and gender of multiply categorizable individuals. *J. Pers. Soc. Psychol.* 85 616–626. 10.1037/0022-3514.85.4.616 14561116

[B30] Kimchi R. (1992). Primacy of wholistic processing and the global/local paradigm: A critical review. *Psychol. Bull.* 112 24–38. 10.1037/0033-2909.112.1.24 1529037

[B31] Klempova B. Liepelt R. (2016). Do you really represent my task? Sequential adaptation effects to unexpected events support referential coding for the joint simon effect. *Psychol. Res.* 80 449–463. 10.1007/s00426-015-0664-y 25833374

[B32] Knoblich G. Sebanz N. (2006). The social nature of perception and action. *Curr. Dir. Psychol. Sci.* 15 99–104. 10.1111/j.0963-7214.2006.00415.x

[B33] Kornblum S. Hasbroucq T. Osman A. (1990). Dimensional overlap: Cognitive basis for stimulus-response compatibility: A model and taxonomy. *Psychol. Rev.* 97 253–270. 10.1037/0033-295X.97.2.253 2186425

[B34] Lee J. Chung D. Chang S. Kim S. Kim S. W. Park H. (2012). Gender differences revealed in the right posterior temporal area during Navon letter identification task. *Brain Imaging Behav.* 6 387–396. 10.1007/s11682-012-9153-8 22370912

[B35] Lee K. Choo H. (2013). A critical review of selective attention: An interdisciplinary perspective. *Artif. Intell. Rev.* 40 27–50. 10.1007/s10462-011-9278-y

[B36] Liepelt R. Wenke D. Fischer R. Prinz W. (2011). Trial-to-trial sequential dependencies in a social and non-social simon task. *Psychol. Res.* 75 366–375. 10.1007/s00426-010-0314-3 21085984

[B37] McClung J. S. Jentzsch I. Reicher S. D. (2013). Group membership affects spontaneous mental representation: Failure to represent the out-group in a joint action task. *PLoS One* 8:e79178. 10.1371/journal.pone.0079178 24278119 PMC3835841

[B38] Memelink J. Hommel B. (2013). Intentional weighting: A basic principle in cognitive control. *Psychol. Res.* 77 249–259. 10.1007/s00426-012-0435-y 22526717 PMC3627030

[B39] Müller B. C. N. Kühn S. van Baaren R. B. Dotsch R. Brass M. Dijksterhuis A. (2011). Perspective taking eliminates differences in co-representation of out-group members’ action. *Exp. Brain Res.* 21 423–428. 10.1007/s00221-011-2654-7 21465413 PMC3102202

[B40] Müller-Oehring E. M. Schulte T. Raassi C. Pfefferbaum A. Sullivan E. V. (2007). Local-global interference is modulated by age, sex and anterior corpus callosum size. *Brain Res.* 1142 189–205. 10.1016/j.brainres.2007.01.062 17335783 PMC1876662

[B41] Mussi D. R. Marino B. F. M. Riggio L. (2015). Experimental psychology Simon effect the influence of social and nonsocial variables on the Simon effect. *Exp. Psychol.* 62 215–231. 10.1027/1618-3169/a000292 26421448

[B42] Navon D. (1977). Forest before trees. The precedence of global features in visual perception. *Cogn. Psychol.* 9 353–383. 10.1016/0010-0285(77)90012-3

[B43] Navon D. (1981). The forest revisited: More on global precedence. *Psychol. Res.* 43 1–32. 10.1007/BF00309635

[B44] Navon D. (2003). What does a compound letter tell the psychologist’s mind? *Acta Psychol.* 114 273–309. 10.1016/j.actpsy.2003.06.002 14670701

[B45] Peterburs J. Liepelt R. Voegler R. Ocklenburg S. Straube T. (2017). It’s not me, it’s you. Differential neural processing of social and non-social nogo cues in joint action. *Soc. Neurosci.* 12 1–11. 10.1080/17470919.2017.1403374 29115181

[B46] Pfister R. Dolk T. Prinz W. Kunde W. (2014). Joint response-effect compatibility. *Psychon. Bull. Rev.* 21 817–822. 10.3758/s13423-013-0528-7 24101572

[B47] Pletzer B. (2014). Sex-specific strategy use and global-local processing: A perspective toward integrating sex differences in cognition. *Front. Neurosci.* 8:425. 10.3389/fnins.2014.00425 25565953 PMC4273628

[B48] Powlishta K. K. (1995). Intergroup processes in childhood: Social categorization and sex role development. *Dev. Psychol.* 31 781–788. 10.1037/0012-1649.31.5.781

[B49] Prinz W. (2015). Task representation in individual and joint settings. *Front. Hum. Neurosci.* 9:268. 10.3389/fnhum.2015.00268 26029085 PMC4428057

[B50] Razumnikova O. M. Volf N. V. (2011). Information processing specialization during interference between global and local aspects of visual hierarchical stimuli in men and women. *Hum. Physiol.* 37 137–142. 10.1134/S0362119711020186

[B51] Roalf D. Lowery N. Turetsky B. I. (2006). Behavioral and physiological findings of gender differences in global-local visual processing. *Brain Cogn.* 60 32–42. 10.1016/j.bandc.2005.09.008 16271817

[B52] Ruys K. I. Aarts H. (2010). When competition merges people’s behavior: Interdependency activates shared action representations. *J. Exp. Soc. Psychol.* 46 1130–1133. 10.1016/j.jesp.2010.05.016

[B53] Sebanz N. Bekkering H. Knoblich G. (2006). Joint action: Bodies and minds moving together. *Trends Cogn. Sci.* 10 70–76. 10.1016/j.tics.2005.12.009 16406326

[B54] Sebanz N. Knoblich G. (2009). Prediction in joint action: What, when, and where. *Top. Cogn. Sci.* 10 70–76. 10.1111/j.1756-8765.2009.01024.x 25164938

[B55] Sebanz N. Knoblich G. Prinz W. (2003). Representing others’ actions: Just like one’s own? *Cognition* 88 B11–B21. 10.1016/S0010-0277(03)00043-X 12804818

[B56] Sellaro R. Dolk T. Colzato L. S. Liepelt R. Hommel B. (2015). Referential coding does not rely on location features: Evidence for a nonspatial joint Simon effect. *J. Exp. Psychol. Hum. Percept. Perform.* 41 186–195. 10.1037/a0038548 25528013

[B57] Simon J. R. (1969). Reactions towards the source of stimulation. *J. Exp. Psychol.* 81 174–176. 10.1037/h0027448 5812172

[B58] Simon J. R. (1990). “The effects of an irrelevant directional cue on human information processing,” in *Stimulus-response compatibility: An integrated perspective. advances in psychology*, eds Proctor R. W. Reeve T. G. (Amsterdam: Elsevier Science Publishers), 31–86.

[B59] Stangor C. Lynch L. Duan C. Glass B. (1992). Categorization of individuals on the basis of multiple social features. *J. Pers. Soc. Psychol.* 62 207–218. 10.1037/0022-3514.62.2.207

[B60] Stock A. Stock C. (2004). A short history of ideo-motor action. *Psychol. Res.* 68 176–188. 10.1007/s00426-003-0154-5 14685855

[B61] Stoet G. (2010). Sex differences in the processing of flankers. *Q. J. Exp. Psychol.* 63 633–638. 10.1080/17470210903464253 20013515

[B62] Stoet G. (2017). Sex differences in the Simon task help to interpret sex differences in selective attention. *Psychol. Res.* 81 571–581. 10.1007/s00426-016-0763-4 26957425 PMC5397428

[B63] Tajfel H. Turner J. C. (1979). “An integrative theory of intergroup conflict,” in *The social psychology of intergroup relations*, eds Austin W. G. Worchel S. (Monterey, CA: Brooks-Cole), 33–47.

[B64] Tsai C. C. Kuo W. J. Hung D. L. Tzeng O. J. L. (2006). A common coding framework in self-other interaction: Evidence from joint action task. *Exp. Brain Res.* 175 353–362. 10.1007/s00221-006-0557-9 16799815

[B65] van der Weiden A. Aarts H. Prikken M. van Haren N. E. M. (2016). Individual differences in action co-representation: Not personal distress or subclinical psychotic experiences but sex composition modulates joint action performance. *Exp. Brain Res.* 234 499–510. 10.1007/s00221-015-4475-6 26525711 PMC4731433

[B66] Welsh T. N. Kiernan D. Neyedli H. F. Ray M. Pratt J. Potruff A. (2013). Joint Simon effect in extrapersonal space. *J. Mot. Behav.* 45 1–5. 10.1080/00222895.2012.746635 23387518

[B67] Witt M. G. Wood W. (2010). Self-regulation of gendered behaviour in everyday life. *Sex Roles* 62 635–646. 10.1007/s11199-010-9761-y

[B68] World Medical Association. (2013). World medical association declaration of helsinki. ethical principles for medical research involving human subjects. *JAMA* 310 2191–2194. 10.1001/jama.2013.281053 24141714

[B69] Yamaguchi M. Wall H. J. Hommel B. (2017). Action-effect sharing induces task-set sharing in joint task switching. *Cognition* 165 113–120. 10.1016/j.cognition.2017.05.022 28535468

